# Mechanisms of vitamin D on skeletal muscle function: oxidative stress, energy metabolism and anabolic state

**DOI:** 10.1007/s00421-019-04104-x

**Published:** 2019-03-04

**Authors:** Katarzyna Patrycja Dzik, Jan Jacek Kaczor

**Affiliations:** Department of Neurobiology of Muscle, Gdansk University of Physical Education and Sport, Kazimierza Gorskiego 1, 80-336 Gdansk, Poland

**Keywords:** Vitamin D, Skeletal muscle, Vitamin D receptor, Mitochondria, Muscle atrophy

## Abstract

**Purpose:**

This review provides a current perspective on the mechanism of vitamin D on skeletal muscle function with the emphasis on oxidative stress, muscle anabolic state and muscle energy metabolism. It focuses on several aspects related to cellular and molecular physiology such as VDR as the trigger point of vitamin D action, oxidative stress as a consequence of vitamin D deficiency.

**Method:**

The interaction between vitamin D deficiency and mitochondrial function as well as skeletal muscle atrophy signalling pathways have been studied and clarified in the last years. To the best of our knowledge, we summarize key knowledge and knowledge gaps regarding the mechanism(s) of action of vitamin D in skeletal muscle.

**Result:**

Vitamin D deficiency is associated with oxidative stress in skeletal muscle that influences the mitochondrial function and affects the development of skeletal muscle atrophy. Namely, vitamin D deficiency decreases oxygen consumption rate and induces disruption of mitochondrial function. These deleterious consequences on muscle may be associated through the vitamin D receptor (VDR) action. Moreover, vitamin D deficiency may contribute to the development of muscle atrophy. The possible signalling pathway triggering the expression of Atrogin-1 involves Src-ERK1/2-Akt- FOXO causing protein degradation.

**Conclusion:**

Based on the current knowledge we propose that vitamin D deficiency results from the loss of VDR function and it could be partly responsible for the development of neurodegenerative diseases in human beings.

## Introduction

**Fig. 1:**
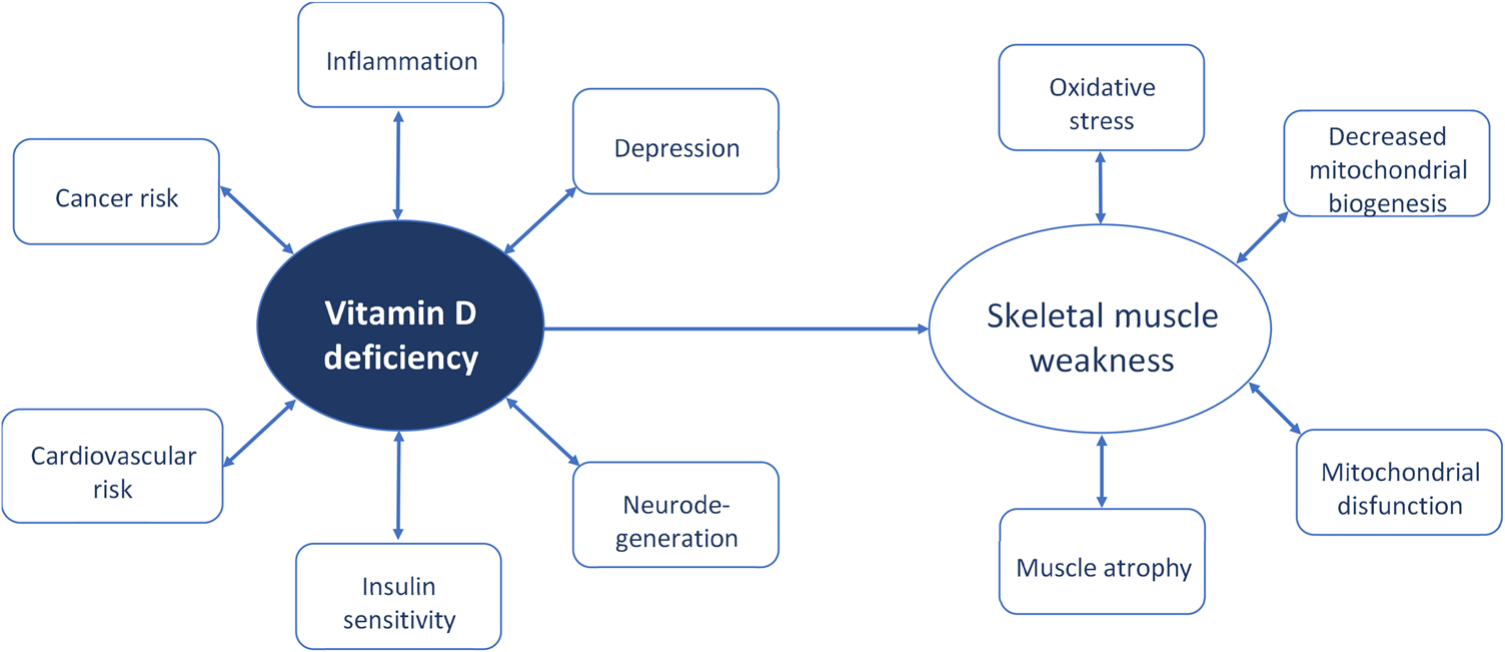
Overview of biological functions of vitamin D with the emphasis on skeletal muscle

The last decade brought a tremendous number of studies on vitamin D function in human body. In fact, those studies have begun in 1822 when the Polish physician Dr. Jedrzej Sniadecki discovered that the lack of sunlight exposure directly contributes to the onset of rickets (Mozolowski 1939). Later, in 1918 Sir Edward Mellanby showed that nutritional intervention with cod liver oil may replace sunlight in the cure and prevention of rickets (Mellanby 1918). Next, Dr. Elmer McCollum et al. officially termed this nutritional factor as vitamin D (McCollum et al. 1922). The discovery of vitamin D receptor (VDR) (Haussler et al. 1969), and confirming its presence in various tissues has opened the mechanistic link between vitamin D and the occurrence of many diseases and disorders such as: obesity, a chronic, low-grade inflammatory state which aids in the pathogenesis of insulin resistance, metabolic syndrome, and type II diabetes mellitus, (McGill et al. 2008), cardiovascular risk (Kunadian et al. 2014), Alzheimer’s disease (Littlejohns et al. 2014), depression (Jhee et al. 2017) and cancer (Garland et al. 2006). The presence of VDR was also confirmed in skeletal muscle tissue (Simpson et al. 1985; Bischoff et al. 2001), thereby the studies on musculoskeletal disorders gained the potential to evaluate the mechanistic properties of vitamin D function (Fig. 1). The aim of this review is to present the latest reports on skeletal muscle function and vitamin D status. The current review provides the evidence that deficiency of vitamin D through oxidative stress and disruption of mitochondrial function may affect the development of skeletal muscle atrophy.

## Vitamin D deficiency

Over the past 2 decades, interest in vitamin D has increased significantly. Requests for serum vitamin D concentration measurements increased between the year 2000 and 2010 by over 80-fold and the number of vitamin D supplements sales has risen several times (Shahangian et al. 2014). Although vitamin D intoxication reports are rare (Galior et al. 2018), it does occur and patients and prescribers should be more cognizant of the proper vitamin D treatment and potential dangers of vitamin D overdose. There has been controversy about what exact 25(OH)D (the sum of 25[OH]D_2_ and 25[OH]D_3_ concentrations) define vitamin D deficiency and sufficiency. The latest guidelines indicate the concentration lower than 20 ng/mL (50 nmol/L) as vitamin D deficiency. The majority of studies that included 25(OH)D concentrations to analyze relations between health and the risk of diseases pointed on higher 25(OH)D concentrations, i.e., in the range of 30–50 ng/mL (75–125 nmol/L) or 40–60 ng/mL (100–150 nmol/L), not on 20 ng/mL (50 nmol/L) as the necessary minimal concentration for human well-being (Pludowski et al. 2018). However, the true vitamin D deficiency should consider its bioavailability and, therefore, its binding to vitamin D binding protein (VDBP). VDBP is the primary vitamin D carrier, binding 85–90% of circulating 25(OH)D and 1,25-dihydroxyvitamin D_3_ and the remaining unbound 25(OH)D is considered bioavailable (either free or bound to albumin). About 10–15% of total 25(OH)D is bound to albumin, in contrast to free 25(OH)D, which accounts for 1% of total circulating vitamin D (Bikle et al. 1986). Since the affinity of albumin to 25(OH)D or 1,25(OH)_2_D_3_ is weaker than that of VDBP, the loosely bound fraction and the free fraction comprise bioavailable 25(OH)D (Brown et al. 2012). There might be tremendous personal differences in bioavailable vitamin D for humans with a genetic mutation for VDBP or in VDBP-KD mouse models were shown to have lower 25(OH)D blood level (Fu et al. 2009; Jones et al. 2014). There are also big racial differences in VDBP in the general population to some degree. A recent study demonstrated that although Black people had lower levels of VDBP and serum 25(OH)D (38.9 ± 0.5 nmol/L) than White people (64.4 ± 0.9 nmol/L) the levels of bioavailable 25(OH)D of Black people was similar to those of White people (2.9 ± 0.1 and 3.1 ± 0.1 ng/mL, respectively) (Powe et al. 2013). Vitamin D overdose may lead to vitamin D toxicity. The clinical manifestations of vitamin D toxicity reported in these cases were a consequence of the hypercalcemia and included nausea, vomiting, muscle weakness, polyuria, nephrocalcinosis, and renal failure (Galior et al. 2018).

## Vitamin D physiology

To be fully active, 25(OH) D_3_ (calcidiol, 25-hydroxycholecalciferol) must be hydroxylated in the C-1 position, producing 1α,25(OH)_2_ D_3_ (calcitriol, 1,25-dihydroxycholecalciferol). It is known that 1α,25(OH)2 D_3_ concentration is regulated by two vitamin D_3_ regulating enzymes, CYP24A1 (cytochrome P450 family 24 subfamily A member 1), and CYP27B1 (25-hydroxyvitamin D-1-α-hydroxylase). In general, excess 1α,25(OH)_2_ D_3_ can be converted to its catabolite form 1α,24,25(OH)_3_ D_3_ via the CYP24A1 enzyme in which 1α,24,25(OH)_3_ D_3_ is less active. In contrast, 25-hydroxyvitamin D-1α-hydroxylase functions to increase 1α,25(OH)_2_ D_3_ synthesis from 25(OH) D_3_ (Srikuea et al. 2016). This enzymatic reactions take place mainly in the kidney, although other cells/tissues express the CYP27B1 and CYP24A1 enzymes as well, particularly in C2C12 cells and mouse primary myotubes (Girgis et al. 2014). Therefore, the expression of the vitamin D_3_ metabolizing enzymes in skeletal muscle suggests the possible local regulation of vitamin D_3_ in this extrarenal tissue (Srikuea et al. 2016). In addition to the regulation of the concentration of the active vitamin D_3_, CYP27B1 is a central regulatory axis of the calcium and phosphate homeostatic systems. CYP27B1 is upregulated by parathyroid hormone (PTH), low Ca^2+^, and low PO_4_^3−^ levels (Omdahl et al. 1972; DeLuca 1974). Therefore, a negative relationship exists between serum 25(OH) D_3_ and serum PTH. The threshold of serum 25(OH) D_3_, where serum PTH starts to rise is about 75 nmol/l according to most studies (Lips 2006).

The study of Abboud and coworkers (Abboud et al. 2013), which examined the concentration and time-dependent effects of calcitriol on the capacity of muscle cells to take up and release 25(OH) D_3_, showed an evidence that skeletal muscle cells indeed contain a mobile pool of 25(OH) D_3_ which accumulates from and returns to the extracellular environment. 25(OH) D_3_ is taken up and retained in the muscle cells by binding to VDBP, which is internalized via membrane megalin and then attached to actin in the cytoplasm, that provide high affinity binding for its specific ligand, 25(OH) D_3_. Interestingly, the early increase in net uptake of 25(OH)D_3_ after a short pre-incubation (3 h) and short further incubation (4 h) with calcitriol was associated with a significant increase in VDBP protein in the C2 myotubes, perhaps providing more intracellular binding sites for 25(OH)D_3_. It is possible that this increase in VDBP might be due to reduced degradation in the cell since the authors observed little VDBP in the incubation medium (Abboud et al. 2018). The study also reports that when C2 cells are differentiated into myotubes, the time-dependent uptake of labelled 25(OH) D_3_ is 2–3 times higher than in undifferentiated myoblasts and osteoblasts. Additionally, they showed that C2 myotubes released only 32% of the previously accumulated 25(OH) D_3_ after 4 h as compared to 60% for osteoblasts, and that muscle uptake and retention of 25(OH) D_3_ are modulated by PTH (Abboud et al. 2017). The authors postulated that if the capacity to hold 25(OH) D_3_ out of the circulation in skeletal muscle is high when vitamin D status is falling in winter, 25(OH) D_3_ might be protected from inactivating activity of CYP24A1 in the liver. Furthermore, they hypothesize that-storage and gradual release from muscles would increase the level of circulating 25(OH) D_3_. This would maintain adequate status during the months when vitamin D supply was low giving skeletal muscles a pivotal role in the maintenance of vitamin D status (Abboud et al. 2013, 2017, 2018). It is important to emphasize that also in this hypothesis VDBP plays an important role in regulating bioavailability of vitamin D yet this time in skeletal muscle cells.

## VDR in musculoskeletal system

Many biological functions of the active form of vitamin D_3_ are mediated by VDR, which is a protein that binds 1α,25(OH)_2_ D_3_ effectively at sub-nanomolar concentrations (Haussler et al. 1997; Dusso et al. 2005). 1α,25(OH)_2_ D_3_ binds to VDR what leads to the conformational changes that allow VDR to interact with its heterodimeric partner, retinoid X receptor (RXR) (Smith et al. 2004). The complex (i.e.1,25D-VDR-RXR) is translocated to the nucleus and binds to vitamin D response elements (VDRE), which ultimately results in activation of transcription (Haussler et al. 1998). Classic VDRE consist of two hexameric direct repeats with a three-nucleotide linker (Umesono et al. 1991; Carlberg et al. 1993). The cell specificity of the actions of VDR and its ligand 1α,25(OH)_2_ D_3_ can be explained in part by VDR’s recognition mode for its genomic binding sites and the tissue-specific differences in the expression of VDR and its key co-factors. Moreover, in contrast to other nuclear receptors such as receptors of cortisol or testosterone, the VDR can bind its genomic targets also in the absence of ligand, i.e. in this respect the functional profile of the VDR is larger than that of its ligand (Polly et al. 2000).

Study on chick myoblasts treated with 1α,25(OH)_2_ D_3_ revealed rapid translocation of VDR from the nucleus to the plasma membrane within 5 min after the addition of 1α,25(OH)_2_ D_3_ (Capiati et al. 2002). The 1α,25(OH)_2_ D_3_-dependent intracellular redistribution of the VDR can be blocked by genistein, herbimycin or colchicine, suggesting the involvement of tyrosine kinase/s and microtubular transport in the relocation of the receptor (Capiati et al. 2002). Studies using a VDR knockout (VDRKO) mouse (Zanello et al. 2004) and a naturally occurring human VDR mutation (Nguyen et al. 2004) unquestionably showed that 1α,25(OH)_2_ D_3_-mediated rapid responses require a functional VDR. Despite that, the VDR has been found also in the plasma membrane in caveolae (Norman et al. 2002); therefore, it has been proposed that the VDR activates nongenomic signalling. Interestingly, the identification of an alternative ligand-binding pocket in the nuclear VDR has allowed to generate by computer docking a receptor conformational ensemble model providing an explanation for the VDR genomic and non-genomic functions (Mizwicki et al. 2004).

The *VDR gene* shows highest expression in metabolic tissues, such as kidneys, bone and intestine, but at least low to moderate expression is found in nearly all other of the approximately 250 human tissues and cell-types (Verstuyf et al. 2010). In situ studies on human skeletal muscles confirm the presence of VDR in this tissue (Bischoff et al. 2001) and documented that expression of VDR is essential for effective uptake of vitamin D by muscle cells (Girgis et al. 2014). Additionally, recent study in VDRKO mouse muscle fibers exposed to calcitriol confirmed that VDR is essential for an uptake of labelled 25(OH) D_3_ (Abboud et al. 2018).

Tanaka and coworkers (Tanaka et al. 2014) using C2C12 and G58 cells demonstrated that myoblasts require downstream signalling from VDR for differentiation into myocytes and that VDR expression is necessary in skeletal muscles for maintaining muscle volume. In addition, it has been presented that VDRKO mice exhibit abnormal skeletal muscle development (Endo et al. 2003). Moreover, serum 25(OH) D_3_ levels and the expression of VDR in muscle cells, as well as testosterone, levels, decline with age (Bischoff-Ferrari et al. 2004), which contribute to developing sarcopenia and muscle weakness (Lips et al. 2010). VDR is located predominantly on the fast-twitch muscle fibers, which respond first in rapid actions, thus it is not surprising that vitamin D sufficiency increases muscle strength and coordination, enabling prevention of falls (Suzuki et al. 2008; Holick et al. 2011).

Ceglia and coworkers (Ceglia et al. 2013) showed that 4-month vitamin D supplementation increased intramyonuclear VDR concentration by 30% in nonexercised *vastus lateralis* muscle in the older, mobility-limited, vitamin D-insufficient women. Although, as mentioned before, VDR is predominantly expressed in fast twitch muscles, a study on human paraspinal, slow twice muscle shows that vitamin D deficiency induces its atrophy and decreases the concentration of intramyonucelar VDR and *VDR gene* expression level (Bang et al. 2018). Also, the study on chronic obstructive pulmonary disease mice model shows that VDR expression in both EDL (*extensor digitorum longus*) and *soleus* muscles was reduced in vitamin D-deficient mice as compared with mice with normal vitamin D levels and that the reduction in VDR expression with vitamin D deficiency was more pronounced in the soleus muscle (− 57%) compared with the EDL muscle (− 37%) (Cielen et al. 2016). This data confirms the relationship between serum vitamin D concentration and intramyonuclear VDR concentration, regardless the type of muscle. However, when the disturbed signalling of 1,25(OH)_2_D_3_ is explored, it must be considered that the deficiency of vitamin D and the loss of the VDR have some similar but partly meaningful consequences.

Although in many studies VDR has been shown to be necessary for vitamin D function, numerous non classic sites have been proven to act as VDRE (Girgis et al. 2013). Also, non-genomic effects of vitamin D, characterized by rapid activation followed by other complex pathways of intracellular signal transduction after binding of 1,25(OH)D_3_ to its non-nuclear receptor (Losel et al. 2003; Girgis et al. 2013; Owens et al. 2015) have been reported. Interestingly not only vitamin D itself, but also essential oils (caraway, coriander, dill, ginger, lemongrass, oregano, spearmint, thyme, turmeric and verveine) exhibit the ability to modulate VDR activity (Bartonkova et al. 2018). Intriguingly, essential oils of turmeric, oregano, dill, caraway, verveine and spearmint augmented the activity of both VDR and glucocorticoid receptor (GR) (Bartonkova et al. 2018). The concentrations of essential oils used in this study are naturally occurring in foods and drinks (Usjak et al. 2017). Non-genomic action of vitamin D, diversity in VDR regulation and the presence of numerous VDRE sites widens the range of possible explanations for the mechanism of vitamin D function in the human body and skeletal muscle.

VDR knockout and vitamin D deficiency conditions seem to clearly indicate negative consequences for skeletal muscle homeostasis. Notwithstanding, the overexpression of VDR seems to have damaging consequences on skeletal muscle as well. The FokI polymorphism of *VDR gene* is a T/C transition in the second exon, resulting in a truncated protein (424aa instead of 427aa) with enhanced transactivation capacity (Whitfield et al. 2001). Two studies in humans suggest that FokI polymorphism is associated with decreased skeletal muscle mass and strength. In particular, Roth and coworkers showed that FokI homozygous men display a low fat-free muscle mass and risk of sarcopenia 2.2-fold higher than controls (Roth et al. 2004). The other study demonstrates that homozygosity for the FokI polymorphism is associated with reduced quadriceps strength as compared with heterozygosity or control patients (Hopkinson et al. 2008). Latest reports show that VDR expression in C2C12 cells is high at the beginning of the differentiation process and is progressively reduced until the cells complete their maturation into myotubes. This observation is consistent with previous data reported that mean intracellular VDR content is higher in undifferentiated than in differentiated cells (Kong et al. 2006). In this regard, VDR down-regulation may represent a condition required to achieve complete myogenic differentiation. The presence of several VDRE in the promoter region of the myogenin *gene* and the demonstration that VDR may directly bind (in absence of the administration of vitamin D) the myogenin promoter support the proposed mechanism of regulation: the hypothesis of a ligand-independent, VDR-mediated, negative regulation of myogenin transcription. This hypothesis is supported by the results showing that animals administered overdosed of vitamin D display an impaired muscle regeneration that is associated with increased VDR expression (Camperi et al. 2017). Therefore, taking into account differences in undifferentiated and matured myotubes, as well as in recovering muscle cells in the manner of VDR requirements it seems that the solution for the skeletal muscle maintenance lays between vitamin D/VDR deficiency and its overexpression. Moreover, there should be a different approach towards vitamin D supplementation for children and adolescents whose muscles are in the development stage, for athletes requiring recovery, and for elderly people.

### Vitamin D relationships to oxidative stress and cellular metabolism in skeletal muscle: data from observational studies

Among many newly discovered functions of vitamin D its involvement in calcium (Ca^2+^) homeostasis seems to be undeniable. Vitamin D regulates calcium absorption in the gut and maintenance of serum calcium and phosphate concentrations (Gil et al. 2018). Vitamin D was shown to be also involved in cellular metabolism of skeletal muscle, yet precise basis for the molecular mechanisms activated by vitamin D in muscles is unclear. Vitamin D action in skeletal muscle affects calcium (Ca^2+^) homeostasis which is an important factor in interplay between cytosol and mitochondria which is involved in muscle energy metabolism (Glancy et al. 2012). Vitamin D, through the activity of its active metabolite, 1α,25(OH)_2_ D_3_, is essential for normal calcium (Ca^2+^) and phosphorus balance and the maintenance of skeletal health (DeLuca 2004; Haussler et al. 2008). It has been shown to play an important role in the regulation of skeletal muscle tone and contraction (Li et al. 2018). Vitamin D deficiency is known to alter muscle contraction kinetics by reducing Ca^2+^ reuptake into the sarcoplasmic reticulum, thereby leading to a prolongation of the relaxation phase of muscle contraction (Rodman et al. 1978; Zittermann 2003). Under physiological conditions mitochondria in skeletal muscle fibers uptake cytoplasmic Ca^2+^ released from the sarcoplasmic reticulum during twitch and tetanic responses (Rudolf et al. 2004). Thus, the experiments on vitamin D-deficient chick muscles demonstrated the alterations in oxidative phosphorylation and an inability of muscle mitochondria to retain Ca^2+^ (Mukherjee et al. 1981). Therefore, vitamin D deficiency may be responsible for inadequate Ca^2+^ uptake by the mitochondria which results in the perturbations of cellular metabolic homeostasis (Sinha et al. 2013).

Latest study of Ryan and coworkers (Ryan et al. 2016) demonstrated increased oxygen consumption rate (OCR) of skeletal muscle cells after treatment with1α,25(OH)_2_ D_3_, indicating vitamin D action in the regulation of mitochondrial oxygen consumption and dynamics. In particular, this study showed that respiration coupled to the generation of ATP was increased, which suggests that vitamin D increases the function of mitochondria in muscle. However, direct treatment of isolated mitochondria with 1α,25(OH)_2_ D_3_ failed to increase OCR suggesting that the effects of 1α,25(OH)_2_ D_3_ on OCR might be VDR-dependent or other extra-mitochondrial biochemical events (Ryan et al. 2016). It is important to mention that the treatment with inactive form of vitamin D, 25(OH) D_3_, did not influence the OCR in isolated mitochondria, which suggested that vitamin D and 25(OH) D_3_ will not be useful in the treatment of muscle weakness unless they are metabolized to 1α,25(OH)_2_ D_3_. In fact, that is operative in the context of vitamin D deficiency where high PTH levels drive the rapid metabolism of 25(OH)D_3_ to 1α,25(OH)_2_ D_3_.

**Fig. 2:**
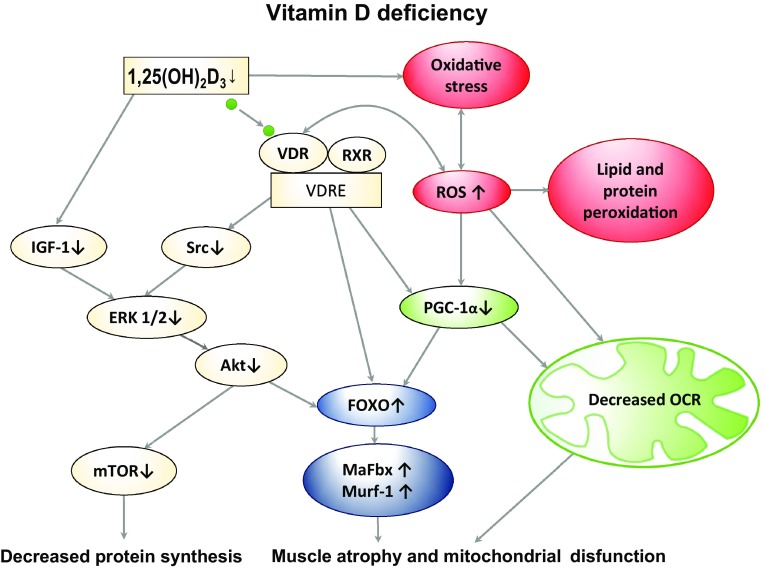
The graphical abstract of the vitamin D action in the skeletal muscle in vitamin D deficiency conditions. Vitamin D deficiency decreases IGF-1 and PGC-1α via VDR—the nuclear receptor. Src/ERK1/2/Akt/FOXO3a signalling cascade triggers the muscle atrophy through Murf-1 and MaFbx. Vitamin D deficiency increases oxidative stress and attenuates mitochondrial biogenesis and function. *Akt* serine/threonine-specific protein kinase, *ERK 1/2* extracellular signal-regulated kinases 1 and 2, *FOXO* forkhead box protein, *IGF-1* insulin-like growth factor 1, *MaFbx* muscle atrophy F-box protein, *mTOR* mammalian target of rapamycin kinase, *MuRF1* muscle ring finger protein, *OCR* oxygen consumption rate, *PGC-1α* peroxisome proliferator-activated receptor gamma coactivator 1-alpha, *ROS* reactive oxygen species, *RXR* retinoid X receptor, *Src* steroid receptor coactivator complex, *VDR* vitamin D receptor, *VDRE* vitamin D response elements

The vitamin D influence on mitochondria was also reported by Sinha and coauthors who showed that treatment of vitamin D deficient humans with cholecalciferol improves the maximal mitochondrial oxidative phosphorylation rate (τ_1/2_PCr and τ_1/2_ADP recovery times were reduced) measured by 31P-NMR spectroscopy (Sinha et al. 2013). Oxidative phosphorylation rate is a function that reflects a composite of mechanisms including mitochondrial number, oxidative enzyme content, mitochondrial components, and vascular supply of substrates and oxygen (Kemp et al. 1993). Moreover, it was reported that the eradication of vitamin D deficiency was associated with an improvement in symptoms of myopathy and fatigue in all participants (Sinha et al. 2013). We found that mitochondrial function was improved in skeletal muscle of patients with low back pain (LBP) supplemented with vitamin D (3200 U/day x 5 weeks). The activity of citrate synthase was approximately 40% higher in the paraspinal muscle after supplementation. Also it was observed higher protein content of PGC-1α, a transcriptional coactivator (unpublished data). The interplay between vitamin D-VDR, reactive oxygen species (ROS) signalling, and the antioxidant system is complex. To the best of our knowledge our group was first demonstrated that vitamin deficiency D increased the cytotoxicity mediated by ROS (Dzik et al. 2018). Taken together, it is very likely that vitamin D deficiency in the long run induces VDR ablation, ROS generation and in consequence deleterious effects on the mitochondrial function, which in turn leads to elevated muscle atrophy (Fig. 2).

Mitochondria not only play an important role in cellular energy metabolism, but they also are a source of ROS. Although mitochondria are not always considered as the main producer of ROS in the cell (NAD(P)H oxidase or xanthine oxidase being able to produce high levels of ROS), the electron transport chain produces ROS continuously (Panel et al. 2018). Therefore, given the data of vitamin D effect on mitochondrial function, the aspect of oxidative stress in skeletal muscle regarding vitamin D deficiency is of great value. Recent study on patients with chronic LBP showed that vitamin D deficiency increases antioxidative enzymes activities (Cu/ZnSOD and GPx) in paraspinal muscle and leads to elevated lipid and protein peroxidation. Moreover, this data demonstrate that five week vitamin D supplementation increases serum vitamin D concentration in LBP patients and decreases oxidative stress in skeletal muscle (Dzik et al. 2018). Similar findings reported increased protein oxidation and nitrosative stress and reduced activities of the antioxidant enzymes (Bhat et al. 2015) as well as increased lipid peroxidation in the muscles of vitamin D-deficient rats (Cielen et al. 2016). Furthermore, another study indicated that rats treated with vitamin D showed reduced tissue damage and attenuated oxidative stress after exhaustive exercise (Ke et al. 2016). This data supports the thesis that vitamin D is involved not only in calcium homeostasis and mitochondrial function but also is responsible for oxidative stress in skeletal muscle.

The exact mechanism which might explain the regulation of oxidative stress via vitamin D is not yet elucidated. As previously mentioned, vitamin D regulates mitochondrial dynamics and function, therefore, it might directly influence the mitochondrial ROS generation. However, it is still a manner of debate if the observed reduction of oxidative stress in skeletal muscle is a result of altered mitochondrial function or it may possibly involve any other capacity of vitamin D action in human body. There are studies demonstrating that vitamin D is a very effective antioxidant. It was shown that vitamin D has a capacity to inhibit zinc-induced oxidative stress in the central nervous system which is 103 times higher than vitamin E analogues (Lin et al. 2005). In addition, another study shows that a vitamin D analogue exerts antioxidant effects by activating the Nrf2-Keap1 antioxidant pathway (Nakai et al. 2014). Nevertheless, vitamin D or vitamin D analogs are able to limit oxidative stress in animals (Hamden et al. 2009; Husain et al. 2009) and humans (Tanaka et al. 2011). Moreover, serum vitamin D concentration correlates with oxidative stress in asthmatic children (Igde et al. 2018) and is associated with adiposity in schoolchildren, suggesting that vitamin D deficiency potentially increases the risk for diseases caused by higher adiposity and oxidative stress (Zhang et al. 2014). For example, adipose tissue releases pro-inflammatory cytokines, resulting in chronic inflammation, which may induce oxidative stress and in consequence leads to muscle damage.

Despite the fact that oxidative stress undeniably has a devastating effect on human body, the importance of ROS and reactive nitrogen species as signals in the skeletal muscle adaptation to exercise is now evident (Merry et al. 2016). There are numerous studies reporting negative consequences of antioxidant supplementation in regard of skeletal muscle function, particularly it was shown to impair mitochondrial biogenesis (Gomez-Cabrera et al. 2008; Ristow et al. 2009), to reduce post-exercise insulin sensitivity (Trewin et al. 2015), as well as to attenuate performance improvements (Braakhuis et al. 2015). In a similar manner, the supraphysiological dose of 1α,25(OH)_2_D_3_ injected into damaged muscle (days 4–7 after BaCl_2_ treatment) delays the regenerative response in muscle namely, decreases satellite cell differentiation, delays regenerative muscle fiber formation, and increases muscular fibrosis (Srikuea et al. 2016). Moreover, as previously mentioned, the study on C2C12 and primary myoblasts clearly shows that vitamin D treatment at supraphysiological dose causes VDR overexpression and impairs their differentiation into mature myotubes (Camperi et al. 2017). These observations suggest that while vitamin D deficiency enhances oxidative stress, overcorrection of vitamin D status may also have a negative impact on skeletal muscle in the same manner as antioxidants while overdosed.

### Vitamin D signalling with anabolic/catabolic pathways

An increasing body of knowledge suggests the involvement of vitamin D in both anabolic and catabolic pathways in skeletal muscle. One of the reasons of muscle wasting results because of an altered balance in the protein degradation and synthesis rates. Thus, there are three major proteolytic pathways described in the skeletal muscle, namely: the ATP-ubiquitin-dependent system, the lysosomal system, and the cytosolic calcium-activated system (Kandarian et al. 2006). Only, the ATP-ubiquitin-dependent system has been shown to be dependent on vitamin D (Bhat et al. 2013). The study on diet-induced vitamin D deficiency in rats reports no alternation in lysosomal and calpain enzyme activities in vitamin D deficiency-induced muscle wasting. However, it shows a significant increase in the enzymes activities of the 20S proteasome catalytic core (Bhat et al. 2013). 20S proteosomal subunit are catalytic part of the 26S proteasome that functions as the key role in nonlysosomal protein degradation (Tawa et al. 1997). Moreover, the study of Bhat and coworkers has been shown an increase in the expression of E2- ubiquitin conjugating enzyme and ubiquitin conjugates in vitamin D deficiency muscle as well as an increase in the expression of 2 muscle-specific E3 ligases. Atrogin-1 also known as MaFbx (muscle atrophy F-box protein) and MuRF1 (muscle ring finger protein) which were increased by twofold in the vitamin D deficient muscle as compared with control (Bhat et al. 2013). It was postulated that Atrogin-1 and MuRF1, which provide substrate specificity in ATP-dependent ubiquitin proteasome pathway (UPP) responsible for intracellular proteolysis are critical for the development of muscle atrophy.

Recent study on mice reports that prolonged, 12 months, vitamin D insufficiency induces characteristics of sarcopenia that include poor anaerobic capacity, lower lean mass, and a trend towards smaller fast twitch fiber cross-sectional area, as well as gait disturbance. Moreover, this study shows that vitamin D insufficient mice also exhibited increased expression of atrophy-associated Atrogin-1 and differential expression of muscle regulation associated miR-26a when compared to mice with normal vitamin D level (Sleeman et al. 2017). This data strongly suggest that vitamin D insufficiency/deficiency is involved in muscular atrophy development, yet the exact mechanism still needs to be explicated.

Hitherto studies postulated that the possible signalling pathway involved in muscle vitamin D function might require steroid receptor coactivator complex (Src), non-receptor tyrosine kinase, which has been shown to activate mitogen-activated protein kinases (MAPK) (Li et al. 2006; Thobe et al. 2006) in various tissues. There are studies that demonstrate fast non-genomic Src activation by vitamin D in various cell types, including skeletal muscle myoblasts (Chappel et al. 1997; Gniadecki 1998; Khare et al. 1999; Buitrago et al. 2001a, b). Furthermore, there is evidence that vitamin D induces rapid association of VDR with Src in skeletal muscle and osteoblastic cells (Buitrago et al. 2000; Vertino et al. 2005). The study of Buitrago and coworkers (Buitrago et al. 2001a, b) reported that the activation of nonreceptor tyrosine kinase Src coincides with a 1,25(OH)_2_ D_3_-induced interaction between Src kinase and VDR in chick muscle cells. Src activation is required for vitamin D-dependent activation of extracellular signal-regulated kinases 1 and 2 (ERK 1/2) and p38 MAPK in skeletal muscle myoblasts (Buitrago et al. 2001a, b, 2006). MAPK signalling is necessary for the maintenance of skeletal muscle mass because inhibition of these signalling cascades elicits muscle atrophy in vitro and in vivo. Particularly, the study on cultured myocytes show that inhibition of ERK1/2 signalling induce myotube atrophy and cause the upregulation of atrophic markers Atrogin-1 and MuRF1 and downregulates the phosphorylation of Akt and its downstream kinases (Shi et al. 2009). Akt along with its downstream signal cascades has been identified as pivotal regulators of muscle hypertrophy by enhancing protein synthesis and concomitant repression of protein breakdown (Sandri et al. 2004; Stitt et al. 2004). It has been reported that 1,25(OH)_2_ D_3_-induced Akt activation in skeletal muscle myoblasts was mediated by Src (Buitrago et al. 2012).

ERK signalling is also suggested to mediate the hypertrophic effects of IGF-I, a muscle hypertrophy factor (Haddad et al. 2004). The IGF-1 is well described as far as it concerns its circulating level. Wei and coworkers have shown that IGF-1 caused an increase in the blood levels of 1,25(OH)_2_D_3_, the hormonally active vitamin D metabolite, by stimulating the expression and activity of the hydroxylase-1α that produces 1α,25(OH)_2_ D_3_ in the kidney (Wei et al. 1998). Moreover, when vitamin D was administered to adult human, IGF-1 serum levels were increased (Ameri et al. 2013). On the other hand, another study reported that 1 year of high-dose vitamin D supplementation did not significantly alter serum IGF-1 among women at high risk in breast cancer (Crew et al. 2015) nor in pre-diabetes patients (Sinha-Hikim et al. 2015).

Recently, Hayakawa and coworkers showed that IGF-1 is not directly affected by 1,25(OH)_2_D_3_ in skeletal muscle. They suggested that vitamin D stimulated IGF-1 production in tissues other than skeletal muscle and that the induced IGF-1 circulates in the blood, and exert hypertrophic effects on muscle tissue or supportive effects on muscle function (Hayakawa et al. 2015). It was reported that IGF-1 signalling induces anabolism, being upregulated during resistance exercise, and this influences the IGF- 1–Akt–FOXO pathway (Perrini et al. 2010; Banerjee et al. 2013; Bonaldo et al. 2013). The major signalling pathways that regulate the size of myofibres are the IGF-1– Akt– FOXO pathway, myostatin, NFκ B and glucocorticoids (Fielding et al. 2011; Bonaldo et al. 2013). In the IGF-1– Akt– FOXO pathway, Akt controls protein synthesis via mTOR, and protein degradation via transcription factors of the FOXO family. FoxO also signals between protein breakdown and synthesis, with FOXO3 playing a role in suppression of protein synthesis, and Akt plays a role in anabolism by suppressing protein breakdown (Bonaldo et al. 2013). FoxO are major regulators of the ubiquitin proteasome system acting by directly regulating muscle-specific E3 ligases.

Recent study showed that VDR signalling enhanced by vitamin D treatment inhibited FOXO1 expression, nuclear translocation, and activity in C2C12 muscle cells. The vitamin D-dependent suppression of FOXO1 activation disappeared when VDR was knocked down. These results suggest that FOXO1 is a major target mediating VDR-null signalling in skeletal muscle including the progression of muscle atrophy (Chen et al. 2016). Hence, FOXO transcription factors are thought to control half of the genes identified in the molecular “common atrophy blueprint” present in different atrophy types (Sandri et al. 2004). Akt, a protein kinase B, which is important in signalling pathways is involved in the protein synthesis and skeletal muscle growth (Schiaffino et al. 2011). Akt blocks the function of the FOXO3 by phosphorylation of conserved residues, leading to their sequestration in the cytoplasm away from target genes (Brunet et al. 1999). Phosphorylated FOXO3a does not translocate to the nuclei, and consequently the expression of MAFbx and MuRF, both target genes of FOXO, are inhibited. It is important to note that elevated PGC-1α content, besides its function in mitochondrial biogenesis, prevents transcriptional activity of FOXO3a (Sandri et al. 2006), therefore the mitochondria might be involved in the atrophy progression. Besides the involvement in the progression of muscle atrophy Akt may also regulate muscle synthesis via mTOR. The study of Salles and coworkers (Salles et al. 2013) 1α,25(OH)_2_D_3_ sensitizes the Akt/mTOR-dependant pathway to the stimulating effect of leucine and insulin, resulting in a further activation of protein synthesis in murine C2C12 skeletal myotubes.

Although vitamin D deficiency has been shown to lead to muscle atrophy both in animals and humans (Endo et al. 2003; Dhesi et al. 2004; Sato et al. 2005; Snijder et al. 2006) the problem of muscle atrophy seems to be more complicated and may not be fully solved with vitamin D alone. While the studies on vitamin D deficiency seem to undoubtedly connect the low level of vitamin D with the progression of muscle atrophy, it is necessary to mention that the propriety to prevent muscle atrophy is not only typical for vitamin D. Catechins and other antioxidants possess the ability to prevent, mitigate, delay, and even treat muscle-related disorders caused by aging and diseases as well (Li et al. 2019). It was reported that epigallocatechin gallate protects the skeletal muscle mitochondria (Oliveira et al. 2016) reduces skeletal muscle oxidative stress in non-obese diabetic rats and restores the content of complex I and voltage-dependent anion selective channel protein 1 (VDAC1) to improve the function of mitochondria (Yan et al. 2012). Epigallocatechin gallate may also decrease the protein degradation rate caused by muscle atrophy, increase the expression of anabolic factors and promote the cross-sectional area of muscle fiber (Mirza et al. 2014; Meador et al. 2015). Catechins were also shown to promote the differentiation of myoblasts (Kim et al. 2017). It is necessary to mention that the changes triggered by catechins involve the same pathways that we previously described to be engaged in vitamin D action in skeletal muscle. Epigallocatechin gallate was shown to promote the phosphorylation of Akt, inhibit the activation of FOXO, prevent nuclear accumulation and reduce the degradation of muscle protein (Bartholome et al. 2010). Epicatechin has been reported to activate Erk1/2 and p38 MAPK, suggesting its potential role in promoting cell survival (Deng et al. 2012). The recent study conducted on human, mice and C2C12 myoblasts demonstrate that the improvement in the glutathione (GSH) status exerts measurable and beneficial effects on both mRNA and protein expression levels of VDBP, VD-25-hydroxylase, VDR as well as PGC-1a/GLUT 4 (Jain et al. 2018). GSH is a major antioxidant and a cofactor of many enzymes in the human body (Franco et al. 2012) and the authors suggest that GSH status positively upregulates the bioavailability of 25(OH)VD. This may explain why consumption of food rich in l-cysteine/methionine and GSH, such as milk and leafy vegetables, can increase the bioavailability of vitamin D and improve the quality of life, while the studies on vitamin D itself are not always consistent.

Intriguingly, glucocorticoids were shown to increase VDR expression, particularly, dexamethasone (Dex) was reported to potentate calcitriol effects by increasing VDR. Treatment of squamous cell carcinoma VII cells with Dex produces an important increase of VDR transcripts. Similar effects have been observed in mouse adipocytes and human breast cancer cell lines. The *VDR* gene contains a number of putative glucocorticoid response elements. Rapid increase in VDR transcript levels may indicate glucocorticoids directly induce VDR de novo transcription (Hidalgo et al. 2010). Also, the study in human monocytes shows that vitamin D enhances glucocorticoid action (Zhang et al. 2013). However, glucocorticoids are known to cause both muscle wasting and decreased bone formation (Ziegler et al. 1998), so the opposite effect to the vitamin D action in skeletal muscle. Vitamin D is considered a true steroid hormone, and like glucocorticoids and gonadal hormones, may exert several immunomodulatory activities (Cutolo et al. 2014). In fact, vitamin D is recognized as an anti-inflammatory agent (Calton et al. 2015), thus vitamin D may influence bioenergetics through inflammatory mechanisms, whereas glucocorticoids rise in the stress condition (Kudielka et al. 2010). Also, our recent study showed that the elevated plasma corticosterone concentration due to chronic stress response was associated with increased oxidative stress in skeletal muscle (Karnia et al. 2018) and the progression of muscle atrophy (data not shown) in rats. Also, cross-sectional analysis of the National Health and Nutrition Examination Survey 2001–2006 report demonstrated that the odds of having vitamin D deficiency were twice as likely in patients who reported glucocorticoids use compared with those without steroid use (Skversky et al. 2011). The different action of vitamin D and glucocorticoids might be due to different concentration, and the right level of vitamin D deficiency and toxicity should be clearly defined in order to alleviate the possible negative glucocorticoid action in skeletal muscle.

## Summary

As summarized in Table 1. recently published data indicates that vitamin D deficiency is associated with lower VDR content, increased oxidative stress and altered the activity of antioxidant enzymes in skeletal muscle. Moreover, it is shown that vitamin D deficiency may induce paraspinal muscle atrophy and decreases the concentration of intramyonucelar VDR and gene expression of VDR. In addition, it is also reported that vitamin D regulates mitochondrial oxygen consumption and dynamics. Namely, vitamin D deficiency decreases oxygen consumption rate and induces disruption of mitochondrial function. Taken together, it is very likely that vitamin D deficiency in the long run induces VDR ablation, ROS generation and in consequence deleterious effects on the mitochondrial function, which in turn leads to elevated muscle atrophy. The possible signalling pathway that triggers the expression of Murf1 and MaFbx (markers of muscle atrophy) may involve Src-ERK1/2-Akt- FOXO. In addition, it should be stressed that the dysfunctions of mitochondrial respiratory chain and dangerous ROS generation are crucial factors in human pathologies, especially in neurodegenerative diseases where muscle atrophy is observed. We assume that vitamin D deficiency results from the loss of VDR function and it could be partly responsible for the development of neurodegenerative diseases in human beings. However, the correction of vitamin D deficiency should be done wisely in order to avoid negative consequences of VDR overexpression and vitamin D toxicity. Vitamin D supplementation should be addressed towards bioavailability of vitamin D and towards personal requirements that may differ between children, athletes, adults and elderly people. Also, the changes in diet in regard of antioxidants, GSH precursors and essential oils supply should be considered as the support for vitamin D treatment.

**Table 1:** Summary of selected studies on the role and the action of vitamin D in skeletal muscle since 2012

Study (ref)	Type of study	Treatment	Main outcome
Buitrago et al. (2012)	Experimnetal study in murine C2C12 skeletal myoblasts	Cells treated with 1 nM 1α,25(OH)_2_D_3_	Vitamin D upregulates Akt through Src, PI(3)K, and p38 MAPK to stimulate myogenesis
Bhat et al. (2013)	Experimental study in rats	Diet-induced vitamin D deficiency	MaFbx and MuRF1 increased by twofold in the vitamin D deficient muscle, increased activity of 20S proteasome catalytic core, induced muscle protein degradation
Ceglia et al. (2013)	Experimental study in vitamin D-insufficient women (aged ≥ 65 years)	Vitamin D insufficient group (22.5 to 60 nmol/L) supplemented with 4 000 (IU/day) for 4 months	Increased intramyonuclear VDR concentration by 30% in nonexercised vastus lateralis muscle
Bhat and Ismail (2015)	Experimental study in rats	Diet-induced vitamin D deficiency	Increased oxidative stress, increased GPx activity, decreased SOD and CAT activities in the rat muscle
Chen et al. (2016)	Experimental study in murine C2C12 skeletal myoblasts	Cells treated with 1α,25(OH)_2_D_3_ (0.01 µM) for 48 h knockdown of VDR	FOXO1 throught VDR signalling causes the progression of muscle atrophy in skeletal muscle, vitamin D deficiency induces insulin resistance
Ryan et al. (2016)	Experimental study in human skeletal muscle cells (hSkMCs)	Cells treated with 1α,25(OH)_2_D_3_ (0.01 µM) for 48 h	Increased oxygen consumption rate
Srikuea and Hirunsai (2016)	Experimental study in male C57BL/6 mice	Supraphysiological (1 µg/kg relative to mouse body weight) dose of 1α,25(OH)_2_D_3_ injected into damaged muscle (days 4–7 after BaCl_2_ treatment)	Decreased satellite cell differentiation, delayed regenerative muscle fiber formation, and increased muscular fibrosis
Camperi et al. (2017)	Experimental study in AH130-bearing rats	Vitamin D intragastrically administrated 80 IU/kg body weight for 7, 14 and 28 days	Impaired muscle regeneration associated with increased VDR expression
	Experimental study in murine C2C12 skeletal myoblasts	Calcitriol (10 nM or 100 nM, supraphisiological dose), starting from the first day of differentiation	Impaired differentiation
Sleeman et al. (2017)	Experimental study in mice	Prolonged diet-induced vitamin D insufficiency (12 months)	Sarcopenia that include poor anaerobic capacity, lower lean mass, and a trend towards smaller fast twitch fiber cross-sectional area, gait disturbance, as well as increased expression of MaFbx and miR-26a
Bang et al. (2018)	Experimental study in women (aged ≥ 60 years)	3 groups: group with normal vitamin D concentration (> 40 ng/mL), vitamin D insufficiency group (20–40 ng/mL) Vitamin D deficient group (< 20 ng/mL)	Vitamin D deficiency induces paraspinal muscle atrophy and decreases the concentration of intramyonucelar VDR and VDR gene expression level
Dzik et al. (2018)	Experimental study in LBP patients	3 groups: vitamin D sufficient group (> 21 ng/mL), vitamin D deficient group (< 20 ng/mL), vitamin D supplemented group (5 weeks, 3200 IU/day)	Vitamin D supplementation decreased oxidative stress in skeletal muscle
Jain et al. (2018)	Experimental study in male C57BL/6J mice	All mice fed vitamin D-deficient diet for 16 weeks. Then divided into 5 groups, diet rich in: saline, olive oil, l-cysteine (5 mg/kg BW), vitamin D (67 IU/kg BW), or l-cysteine + vitamin D	Upregulation of PGC-1a/GLUT 4 and GSH status in muscle of mice supplemented with l-cysteine + vitamin D group in comparison with vitamin D alone supplemented mice
	Experimental study in overweighted adolescent boys and girls ages 14–17	No treatment	Link between vitamin D deficiency and a reduction in glutathione (GSH)
	Experimental study in murine C2C12 skeletal myoblasts	Cells treated with 2 h with l-cysteine (0–300 lM) for 2 h, followed by treatment for 22 h with cholecalciferol or 25(OH)VD (10 nM)	GSH deficiency causes downregulation of PGC-1a, VDR, GLUT 4

## Future directions

Given the important action of vitamin D on skeletal muscle tissue, a better understanding of the mechanisms involved in muscle atrophy is needed. In particular, there is a great need of a new insight into VDR expression and activation, biogenesis and the function of mitochondria as well as signalling pathways associated with progressive muscle atrophy in vitamin D deficiency. On the other hand, beneficial effect of normalized serum vitamin D concentration should be explored in regard to muscle aerobic energy metabolism, oxidative stress and prevention of muscle atrophy. Even more, we suppose that supplementation with vitamin D to sufficient serum vitamin D level will: reduce ROS overproduction, increase *VDR gene* expression and protein content, improve mitochondrial function and inhibit the atrophy of muscle. Finally, the broadened knowledge about vitamin D mechanism(s), may contribute to the reduced progression of neurodegenerative diseases in humans.

## Abbreviations

Akt: Serine/threonine-specific protein kinase
Cu/ZnSOD: Copper/zinc-dependent dismutase
CS: Citrate synthase
CYP24A1: Cytochrome P450 family 24 subfamily A member 1
CYP27B1: 25-hydroxyvitamin D-1-α-hydroxylase
ERK 1/2: Extracellular signal-regulated kinases 1 and 2
FOXO1: Forkhead box protein O1
FOXO3: Forkhead box protein O3
GPx: Glutathione peroxidase
GR: Glucocorticoid receptor
IGF-1: Insulin-like growth factor 1
LBP: Low back pain
MaFbx: Muscle atrophy F-box protein
MAPK: Mitogen-activated protein kinases
MnSOD: Manganese-dependent superoxide dismutase
mTOR: Mammalian target of rapamycin kinase
MuRF1: Muscle ring finger protein
OCR: Oxygen consumption rate
PGC-1α: Peroxisome proliferator-activated receptor gamma coactivator 1-alpha
PTH: Parathyroid hormone
PLIF: Posterior lumbar interbody fusion
ROS: Reactive oxygen species
RXR: Retinoid X receptor
Src: Steroid receptor coactivator complex
VDBP: Vitamin D binding protein
VDR: Vitamin D receptor
VDRE: Vitamin D response elements
